# Src and Abl as Therapeutic Targets in Lung Cancer: Opportunities for Drug Repurposing

**DOI:** 10.3390/ph18101426

**Published:** 2025-09-23

**Authors:** Raquel Ramos, Carlos Sousa, Nuno Vale

**Affiliations:** 1 PerMed Research Group, RISE-Health, Faculty of Medicine, University of Porto, 4200-319 Porto, Portugal; raquel_ramos00@hotmail.com (R.R.); carlos.sousa@unilabs.com (C.S.); 2 RISE-Health, Department of Pathology, Faculty of Medicine, University of Porto, 4200-319 Porto, Portugal; 3 Molecular Diagnostics Laboratory, Unilabs Portugal, Centro Empresarial Lionesa Porto, 4465-671 Leça do Balio, Portugal; 4 Laboratory of Personalized Medicine, Department of Community Medicine, Health Information and Decision (MEDCIDS), Faculty of Medicine, University of Porto, 4200-450 Porto, Portugal

**Keywords:** personalized medicine, drug repurpose, tyrosine kinase inhibitors, Src, Abl, saracatinib, imatinib, PP2, nilotinib, tirbanibulin

## Abstract

Personalized medicine has gained an important relevance over the years with the development of targeted therapies, especially in cancer, adapted to the individual molecular tumour profiles. Accordingly, drug repurposing arises as a powerful strategy to identify and use drugs already approved for other conditions, offering advantages in terms of cost, development time, and safety. Src and Abl tyrosine kinases have been investigated as potential targets in oncology, being frequently implicated in tumour development and progression by promoting cell proliferation, migration, and angiogenesis. This review aims to provide a comprehensive overview of five tyrosine kinase inhibitors—saracatinib, imatinib, PP2, nilotinib and, tirbanibulin—that act on Src and/or Abl. Their mechanisms of action, original therapeutic indications, and potential for repurposing in other diseases, such as lung cancer, will be discussed. Although clinical data for these drugs in lung cancer remain limited, preclinical and clinical studies suggest promising therapeutic potential, particularly in specific molecular subtypes. Overall, this review highlights the therapeutic potential of Src and Abl inhibitors beyond their original contexts and supports their possible role in lung cancer therapy, considering the disease’s high heterogeneity and the growing applicability of personalized medicine.

## 1. Introduction

**Figure 1:**
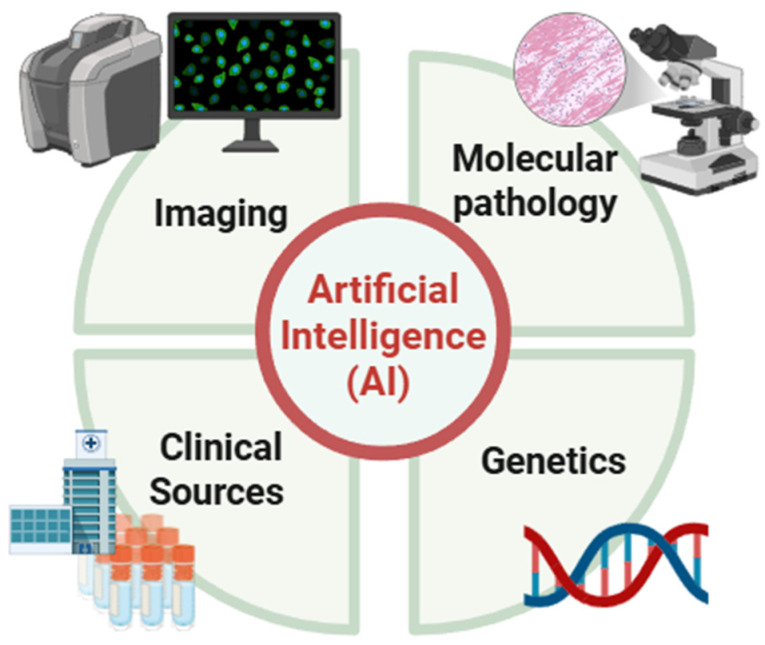
Integration of Artificial Intelligence (AI) in Personalized Medicine (PM) to support data analysis from imaging, molecular pathology, genetics, and other clinical sources as biobanks. Available online: http://biorender.com/ (accessed on 17 June 2025).

Personalized medicine (PM) has gained increasing relevance over the years, driven by advancements in medical methodologies and scientific knowledge. As a result, greater emphasis has been placed on understanding genetic and molecular details, as well as how these molecular processes interact in the entire organism. This has been facilitated by various technologies capable of analyzing data from imaging, molecular pathology, genetics, and clinical sources such as biobanks. Currently, artificial intelligence (AI) plays a significant role in PM by enhancing data analysis, helping in the identification of novel therapeutic targets, and predicting treatment responses more effectively (Figure 1) [1,2]. PM succeeds conventional treatment by tailoring the best therapeutic approach for each patient based on their biological characteristics, thereby reducing the risk of side effects and improving therapeutic response [3]. While this research area is relevant to many diseases, cancer is one of those that stands to benefit the most from PM. Cancer is a heterogeneous disease that can be modulated by genetic, molecular, cellular, environmental, and socioeconomic factors. Moreover, the complexity of the stage and the interpatient variability are the main challenges faced by health professionals. Consequently, the integration of PM in this field is a valuable asset for identifying targeted molecular alterations and enabling individualized treatment, while considering inter- and intra-tumour variability, the immune environment, and each patient’s lifestyle and comorbidities [3,4,5]. Some examples of target genetic mutations include EGFR in lung cancer, which responds to gefitinib and erlotinib therapy, and the Bcr-Abl fusion gene in chronic myelogenous leukemia, which is effectively treated with imatinib [6,7,8]. Other genetic alterations that can be used for personalized and targeted treatment are PIK3CA, ERBB2, FGFR, BRAF, and NTRK mutations or fusions [3].

Apart from PM, drug repurposing is also a growing field for the discovery of new therapies. The development of a new drug is time-consuming, challenging, and expensive, typically taking 10 to 15 years of research, with approximately 90% of drugs ultimately failing to gain US Food and Drug Administration (FDA) approval [9]. Consequently, drug repurposing aims to identify new therapeutic targets or medical conditions for which existing drugs—already approved and/or with established safety profiles—were not previously indicated. This approach can reduce drug development time by 3 to 12 years, allowing researchers to bypass early development stages and proceed directly to preclinical and clinical testing. In some cases, the drug can even advance directly to phase II clinical trials [9,10]. This technique offers several advantages, primarily reducing the risk of failure from a safety perspective. It also shortens the overall drug development timeline and is generally more cost-effective, although the cost can vary depending on the stage and development process of the repurposing candidate [11]. Nowadays, with the development of technology and new methodologies, drug repurposing has gained importance, and several drugs are being repurposed for different conditions [10]. As in PM, cancer is one of the most attractive diseases for drug repurposing. Despite the significant advances in chemotherapy, radiotherapy, surgical, and immunological therapies, the clinical efficacy of several drugs remains a problem due to resistance and toxic side effects. Consequently, drug repurposing is a promising strategy for this disease, considering its shorter time, cost-effectiveness, and safety [12]. Truly, in the field of oncology, drug repurposing is, in itself, not a novel concept [13]. Over the past century, numerous drugs and chemical agents have been approved by the FDA for the treatment of various cancers. Thalidomide, originally developed as a sedative, was repurposed and approved in 1998 for the treatment of multiple myeloma. Other notable examples include arsenic trioxide, a known poison, and all-trans retinoic acid, a metabolite of vitamin A. Both were approved in 2000 for the treatment of acute promyelocytic leukemia [14,15,16,17,18]. Nonetheless, despite the many advantages of drug repurposing in cancer treatment, certain classes of drugs may present greater challenges and may require reformulation or dose adjustment to effectively address the new therapeutic target [11]. 

Despite these challenges, many clinical trials are currently underway or have been completed, exploring the use of repurposed drugs for cancer treatment or prevention. Table 1 summarizes these ongoing and completed trials across various cancer types.

In general, the process of drug repurposing follows three main phases before advancing a candidate through the development pipeline. First, a potential compound is selected for a new therapeutic use based on a scientific hypothesis. Next, its biological effects are investigated using preclinical models to understand its mechanism of action. Finally, if adequate safety data exist from previous phase I trials for the original use, the drug proceeds to phase II clinical studies to assess its effectiveness in the new disease context [11]. Among these steps, understanding the drug’s mechanism of action and molecular targets is particularly crucial. In cancer, numerous signalling pathways are dysregulated, representing important therapeutic opportunities. Notably, Src and Abl are non-receptor tyrosine kinases that play essential roles in regulating cell proliferation, migration, survival, and angiogenesis. These kinases are functionally interconnected, as Abl can be activated downstream of Src and other receptors, contributing to enhanced cancer cell invasion and metastasis. Thus, their aberrant activation has been implicated in the development and progression of several cancers, including lung cancer, making them attractive targets for therapeutic intervention and drug repurposing research [20,21]. 

In this article, we provide an in-depth review of the biological mechanisms of Src and Abl kinases and their involvement in cancer development and progression. Furthermore, we present a comprehensive analysis of five tyrosine kinase inhibitors—saracatinib, imatinib, PP2, nilotinib, and tirbanibulin—that target Src and/or Abl. This review explores their mechanisms of action, original therapeutic indications, and emerging potential for drug repurposing in other diseases, with a particular focus on lung cancer.

## 2. Src and Abl—Non-Receptor Tyrosine Kinases

**Figure 2:**
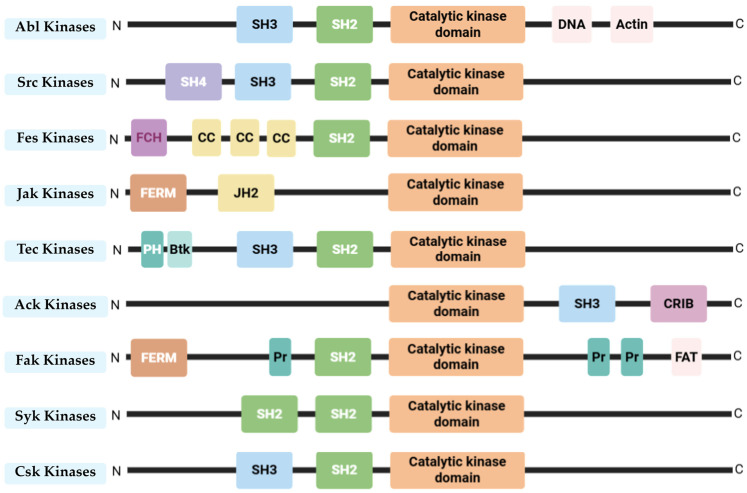
Structure and functional domains of non-receptor tyrosine kinase (NRTKs) families, including Abl, Src, Fes, Jak, Tec, Ack, Fak, Syk, and Csk. N—Amino terminus; C—Carboxy terminus; SH2: SRC Homology 2 domain; SH3—SRC Homology 3 domain; SH4—SRC Homology 4 domain; Catalytic kinase domain (also known as SH1 domain); DNA—DNA binding domain; Actin—Actin binding domain; FCH—Fes/Fer/Cdc-42-Interacting Protein homology domain; CC—Coiled coil motif; FERM—Four-point-one, ezrin, radixin, moesin domain; JH2—Janus homology domain 2 (also known as pseudo kinase domain); CRIB—Cdc42/Rac-interactive domain; PH—Pleckstrin homology domain; Btk—Btk-type zinc finger motif, Pr—Proline rich region, FAT—Focal-adhesion targeting domain.

Protein tyrosine kinases (PTKs) play a crucial role in regulating various cellular processes and signalling pathways. However, their abnormal expression and activation disrupt the cell cycle, differentiation, survival, proliferation, and apoptosis, resulting in disease progression and drug resistance, particularly in cancer [22]. PTKs are divided into two main categories: receptor tyrosine kinases (RTKs) and non-receptor tyrosine kinases (NRTKs). While both types are involved in similar cellular signalling processes, they differ greatly in their structural composition. RTKs are embedded in the cell membrane and function as molecular conduits, relaying external signals from molecules such as growth factors, hormones, and cytokines into the cell and ultimately to the nucleus, being activated by ligand binding. On the other hand, NRTKs are located inside the cell, either in the cytoplasm or the nucleus, as they lack extracellular and transmembrane segments. In the cytoplasm, their main function is to transmit intracellular signalling pathways triggered by the activation of membrane-associated receptors. This subclass of PTKs operates through a more intricate activation mechanism that relies on protein–protein interactions, facilitating transphosphorylation events [23,24]. Furthermore, NRTKs are classified into nine distinct subfamilies, determined by the high degree of sequence similarity within their highly conserved kinase domains [24]. Figure 2 illustrates the various NRTK classes along with the domain organization characteristic of each subfamily.

As described, Src and Abl are part of tyrosine kinase families and are both involved in regulating cell functions, including survival, proliferation, differentiation, migration, apoptosis, and angiogenesis. However, their roles in cancer development and progression have also been confirmed [25,26]. 

### 2.1. Src

**Figure 3:**
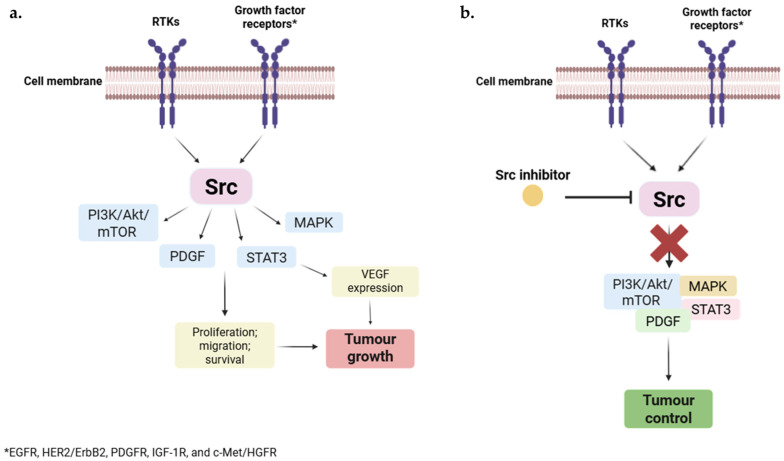
Role of Src in tumour growth and the effect of Src inhibition. (**a**) The activation of receptor tyrosine kinases (RTKs) and other growth factor receptors, such as EGFR, HER2/ErbB2, PDGFR, IGF-1R, and c-Met/HGFR, stimulates Src kinase, leading to activation of signalling pathways (PI3K/Akt/mTOR, MAPK, STAT3, and PDGF). These pathways lead to the production of VEGF and other growth factors, promoting angiogenesis and increasing the proliferation, migration, and survival of cancer cells, ultimately leading to tumour and metastasis progression. (**b**) Inhibition of Src activity using a Src inhibitor blocks the downstream signalling pathways, resulting in a reduction in proliferation, migration, and survival of cancer cells, which leads to tumour control.

Src family of protein tyrosine kinases (SFKs) can be activated by RTKs and is composed by nine members—c-Src; Yes; Fyn; Fgr; Lyn; Hck; Lck; Blk; and Yrk—where c-Src (Src) was the first protein kinase to be described as capable of phosphorylating tyrosine residues [27]. Src, like all members of SFKs, is involved in several biological processes. Specifically, Src is associated with cytoskeletal reorganization and neuronal differentiation, and interacts with growth factor receptors, cell–cell adhesion receptors, integrins, and steroid hormone receptors [25,28]. Src is highly explored in the context of cancer, as it is involved in the activation of several key oncogenic signalling pathways, including the PI3K/Akt/mTOR, MAPK, STAT3, and PDGF signalling pathways. Moreover, Src frequently interacts with multiple growth factor receptors, such as EGFR, HER2/ErbB2, PDGFR, IGF-1R, and c-Met/HGFR, further enhancing downstream signalling and transcriptional activity that drive tumour growth and disease progression. Furthermore, Src plays a critical role in metastasis by regulating key processes such as cell migration, adhesion, and invasion. It facilitates the disruption of cell–cell adherent junctions and promotes angiogenesis by stimulating VEGF expression, particularly under hypoxic conditions. Consequently, Src inhibition arises as a promising possibility for metastasis suppression and tumour control (Figure 3) [27,29]. Specifically, Src was already described in several cancers, such as pancreatic cancer, breast cancer, lung cancer, prostate cancer, and head and neck squamous cell carcinoma [30]. However, the development of effective Src inhibitors continues to face significant challenges, largely due to the lack of specific biomarkers capable of predicting patient response, a limitation driven by the complex nature of Src signalling [21]. 

### 2.2. Abl

The Abl family comprises Abl1 (c-Abl) and Abl2 (Abl-related gene, ARG), and, like Src, both stimulate several signalling pathways related to the control of cell growth, survival, migration, and adhesion, being crucial for maintaining homeostasis. Nonetheless, they are also involved in cancer progression due to their abnormal activation. Once activated, they can reorganize the cytoskeletal network, influencing cell motility, adhesion, and migration. These changes play a crucial role in driving epithelial–mesenchymal transition (EMT) and other key steps in the metastatic cascade [26,31]. Specifically, c-Abl is usually inactivated in human cells, being activated by extracellular and intracellular stimuli, such as DNA damage, autophosphorylation, and reactive oxidative species production [32]. In addition, it is associated with various nuclear proteins that participate in DNA repair, thereby contributing to DNA damage response (DDR). The c-Abl protein can move between the cytoplasm and the nucleus, serving as a crucial link in cell signalling. It transmits signals originating from activated tyrosine kinases directly to the nucleus, where it helps initiate specific DNA repair mechanisms [32]. 

**Figure 4:**
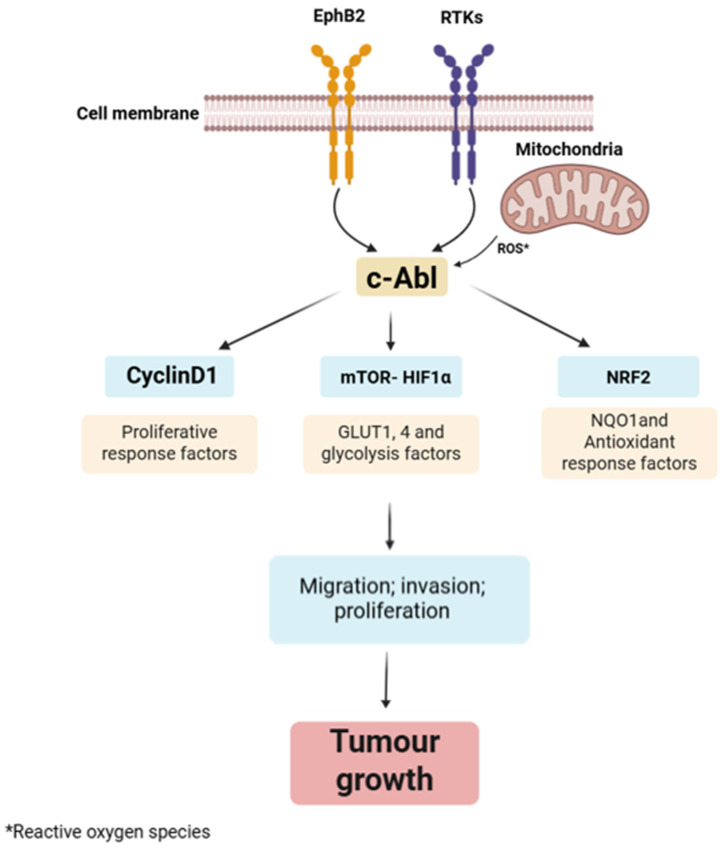
Role of c-Abl (Abl1) in tumour progression. c-Abl is activated by upstream signals from receptor tyrosine kinases (RTKs), EphB2 receptors, and mitochondrial-derived reactive oxygen species (ROS). Upon activation, c-Abl regulates key downstream pathways: Cyclin D1, mTOR–HIF1α, and NRF2. Together, these pathways enhance tumour cells’ proliferation, migration, and invasion, contributing to cancer development and progression.

The first oncogenic link to Abl1 was identified in the Bcr-Abl1 fusion, typical of chronic myeloid leukemia (CML) [33]. However, both Abl1 and Abl2 have also been implicated in solid tumours. Abl1 activity has been observed in lung, kidney, and colorectal cancers, where it regulates signalling pathways such as Cyclin D1 and mTOR-HIF1α. Additionally, through activation of NRF2, it promotes the expression of NQO1 and other antioxidant response genes (Figure 4) [26].

## 3. Src and Abl Inhibitors

As stated earlier, Src and Abl play a crucial role in cellular processes. Nonetheless, their aberrant expression can lead to cancer and, consequently, their inhibition is one of the therapeutic approaches to control tumour growth [34]. 

As previously mentioned, Src belongs to the family of small-molecule protein kinases. According to recent studies, inhibitors targeting these kinases can be classified into six main groups: Type I inhibitors bind competitively to ATP at the kinase’s active conformation; Type II inhibitors also compete with ATP but bind to the kinase in its inactive form; Type III inhibitors are allosteric, attaching adjacent to the ATP-binding pocket; Type IV inhibitors are bivalent and bind at a site distant from the ATP-binding region; Type V cover two kinase domain regions, acting as bivalent modulators; finally, Type VI inhibitors form covalent bonds with the kinase, resulting in irreversible inhibition [35]. Specifically for Src, PP2 is an ATP-competitive Src inhibitor that links to the active form of Src, being studied for cancer treatment [36]. Beyond cancer, inhibition of Src can be useful in other conditions, such as skin conditions. An example of another Src inhibitor is tirbanibulin, approved by FDA for the topical treatment of actinic keratosis on the face or scalp [37]. 

Imatinib is a first-generation, non-allosteric Abl inhibitor that revolutionized the treatment of CML. Nonetheless, the resistance to this drug led to the design of alternative Abl inhibitors that are more specific for this kinase in the treatment of leukemias and solid tumours. Some of the non-allosteric Abl kinase inhibitors are nilotinib, dasatinib, and busotinib. However, these non-allosteric inhibitors are characterized by poor specificity for Abl kinase [38]. Consequently, a new generation of Abl inhibitors—known as allosteric inhibitors—has demonstrated significant efficacy in cancer treatment due to their high specificity for the Abl kinase. Despite their advantages, identifying allosteric sites remains a major challenge, as these sites are often poorly accessible using conventional experimental approaches. Nevertheless, discovering and characterizing these sites is essential for developing more specific and effective Abl inhibitors [39]. 

Beyond imatinib, resistance to other Src and Abl inhibitors remains a clinical problem. Known mechanisms of resistance include mutations within the Bcr-Abl kinase domain, overexpression of Bcr-Abl, and activation of alternative signaling pathways, such as the PI3K/AKT, MAPK, and JAK/STAT pathways. Combining TKIs with immunotherapies, such as anti-PD-1 and anti-CTLA-4 antibodies, developing new Bcr-Abl inhibitors like asciminib (which binds to a distinct region of Bcr-Abl), and exploring epigenetic therapies are being studied as possible strategies to address these mechanisms of resistance [40]. Similarly, mutations in Src can reduce inhibitor affinity and promote drug resistance. Moreover, a study has shown that these mutations can promote Focal Adhesion Kinase (FAK) phosphorylation, thereby increasing cell proliferation and activating the Src-FAK-Grb2-Erk signalling pathway. This activation further stimulates MAPK/ERK and Src/IL-6/STAT3 pathways, ultimately driving transcription of pro-invasive genes, including matrix metalloproteinases (MMPs) [41,42,43]. A relationship between Src and other oncogenic drivers, such as ALK and BRAF, has been reported by some research groups. Therefore, combination therapy with other agents targeting those genes or downstream effectors can be a possible solution to overcome Src resistance [44,45]. 

Although Abl and Src are distinct kinases, research indicates that Abl plays a key role as a substrate in Src signalling within normal cells. Additionally, due to their structural similarities, many Src inhibitors also exhibit strong inhibitory effects on Abl. This cross-reactivity can be advantageous in addressing drug resistance. Examples of dual Src/Abl inhibitors are saracatinib and dasatinib, the latter of which is used in Europe to treat imatinib-resistant CML [34,46]. More specifically, a study already showed that Src family kinases can be activated by Bcr-Abl independently of Bcr-Abl’s kinase function, and once active, these Src kinases can phosphorylate Bcr-Abl [47]. Therefore, at therapeutic level, the dual inhibition may be advantageous by blocking complementary signaling pathways and having a better activity against TKI-sensitive and resistant cancer cells, compared to the ones that only target one kinase. As referred, dasatinib is a clinical example that is active against both imatinib-sensitive and resistant CML [48]. 

### 3.1. Saracatinib

**Figure 5:**
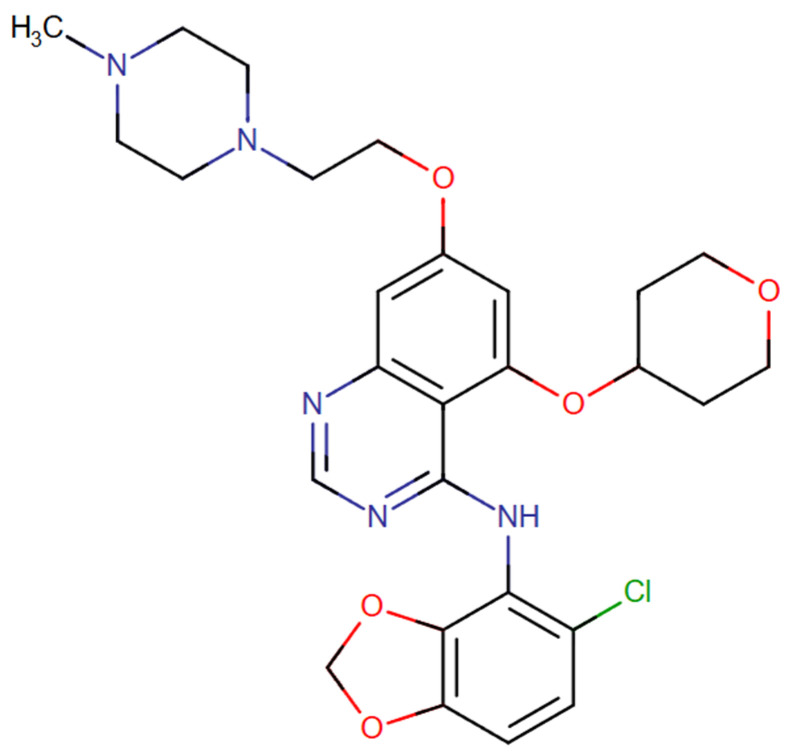
Chemical structures of saracatinib. Available online: https://go.drugbank.com/ (accessed on 21 July 2025).

Saracatinib (AZD0530), N-(5-chloro-1,3-benzodioxol-4-yl)-7-[2-(4-methylpiperazin-1-yl)ethoxy]-5-(tetrahydro-2H-pyran-4-yloxy) quinazolin-4-amine (Figure 5) is a dual c-Src/Abl kinase inhibitor developed by AstraZeneca. It exhibits nanomolar potency, with IC_50_ values of 2.7 nM for Src and 30 nM for Abl [34,49,50]. Saracatinib is classified as a type II Src/Abl inhibitor, as it binds to the inactive conformation of the protein and extends into an adjacent hydrophobic pocket, thereby enhancing its binding affinity and selectivity [34,51]. This drug was first developed for the treatment of solid tumours, targeting Src, completing several preclinical and clinical studies [34]. Beyond cancer, saracatinib was also evaluated in Alzheimer’s disease (AD) [52,53].

Regarding cancer, several preclinical and clinical trials were performed in different kinds of tumours, such as breast, prostate, lung, and colorectal cancer, testing saracatinib alone or in combination with other chemotherapeutic agents. For example, a combination of saracatinib with tamoxifen has been shown to be capable of reducing protein levels related to tumour progression and preventing treatment-acquired hormone resistance, in breast cancer cell lines [54]. Although phase I clinical trials showed some promising outcomes, with a defined well-tolerated dose up to 125/175 mg, multiple phase II studies revealed that the drug had limited effectiveness and was associated with numerous side effects in most cancer types, namely leukopenia, neutropenia, arthralgias, rash, and renal failure [54,55]. Interestingly, a 2014 phase II trial involving platinum-treated non-small cell lung cancer (NSCLC) patients was the only one to report favourable outcomes, including mild side effects, partial tumour regression, and disease stabilization. The findings suggested the existence of an unidentified subgroup of NSCLC patients who may respond to saracatinib. Supporting this, a 2015 preclinical study showed that saracatinib’s effectiveness varies based on specific genetic mutations. Cells with the EGFR T790M mutation and resistance to erlotinib responded well to a combination of saracatinib and cetuximab [56,57]. These findings suggest that the therapeutic efficacy of saracatinib varies depending on the cancer subtype, highlighting the importance of identifying reliable biomarkers to select patients who are most likely to benefit from the treatment [54].

### 3.2. Imatinib

**Figure 6:**
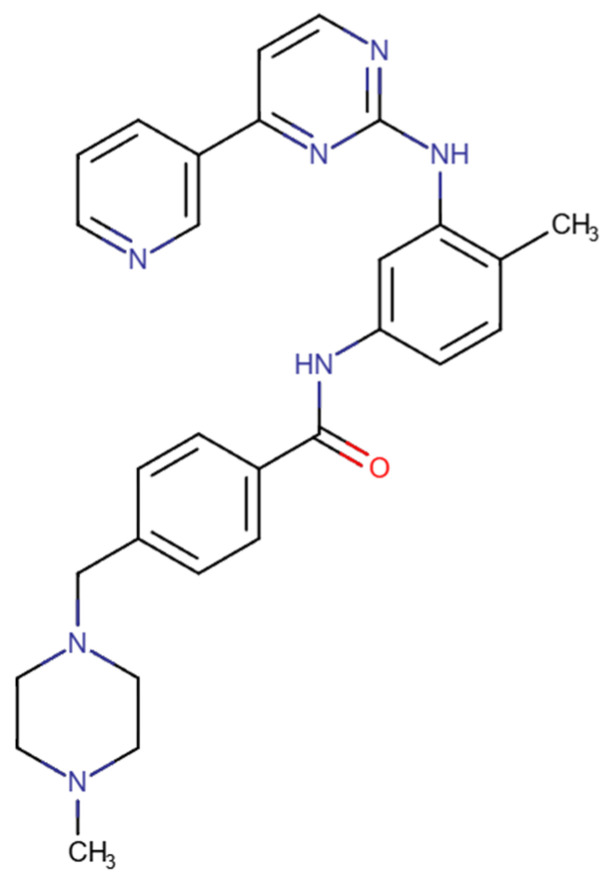
Chemical structures of imatinib. Available online: https://go.drugbank.com/ (accessed on 21 July 2025).

Imatinib (Figure 6) is a tyrosine kinase inhibitor that targets the Bcr-Abl enzyme by competing with ATP for its binding site, thereby inhibiting phosphorylation of protein substrates involved in signal transduction and cell proliferation. Imatinib inhibits Abl with a reported half maximal inhibitory concentration (IC_50_) ranging from 25 to 200 nM [58,59]. This drug was the first tyrosine kinase inhibitor (TKI) approved for clinical treatment of CML, being the first-line treatment in these patients and inducing cell apoptosis and proliferation in those expressing Bcr-Abl, and a daily dose of 400 mg is considered the standard for chronic-phase chronic myeloid leukemia [60,61]. Although not through the blocking of Abl, imatinib is also approved for gastrointestinal stromal tumours (GISTs), characterized by activating mutations in receptor tyrosine kinase genes (most commonly in c-KIT) that increase the proliferation of tumour cells [62]. 

Despite the approval of imatinib for the two types of cancer mentioned above, several studies are being conducted to investigate the potential repurposing of this drug for other cancers, such as lung cancer. Studies suggest that imatinib may be efficient in both NSCLC and small cell lung cancer (SCLC) [63]. Some in vitro studies in SCLC demonstrate encouraging results with imatinib, both as monotherapy and in combination, showing anti-tumoural activity by inhibiting cell growth and tumour angiogenesis, particularly by inhibiting c-Kit signalling. Specifically, in SCLC cell lines, combined treatment with imatinib and vitamin K2, as well as a combination of imatinib with irinotecan, appears to produce favorable therapeutic outcomes [64,65,66]. On the other hand, another study showed opposite results, suggesting that imatinib does not appear to have a therapeutic effect in SCLC [67]. Concordant with this last study, phase II clinical trials results demonstrated no antitumour activity in SCLC patients treated with imatinib targeting KIT receptor [68,69,70,71]. In NSCLC, preclinical studies suggest a favorable effect of imatinib in controlling tumour growth. An in vitro study using Lewis Lung cancer (LLC) cells, which is histologically classified as a type of NSCLC [72], showed that imatinib was capable of affecting tumour growth, by reducing the number of metastasis and decreasing the percentage of M2-like macrophages [73]. Other studies have shown that the A549 cancer cell line is sensitive to imatinib and that this drug exerts an inhibitory effect on the cells. Similarly, several imatinib analogues have also demonstrated strong cytotoxic activity [74]. Using the same cell line, another study also showed that imatinib can reduce the proliferation-stimulating effect of cancer-associated stromal fibroblasts [75]. Nonetheless, like SCLC, the clinical trial results of imatinib in NSCLC patient therapy have not shown encouraging results [76,77].

### 3.3. Nilotinib

**Figure 7:**
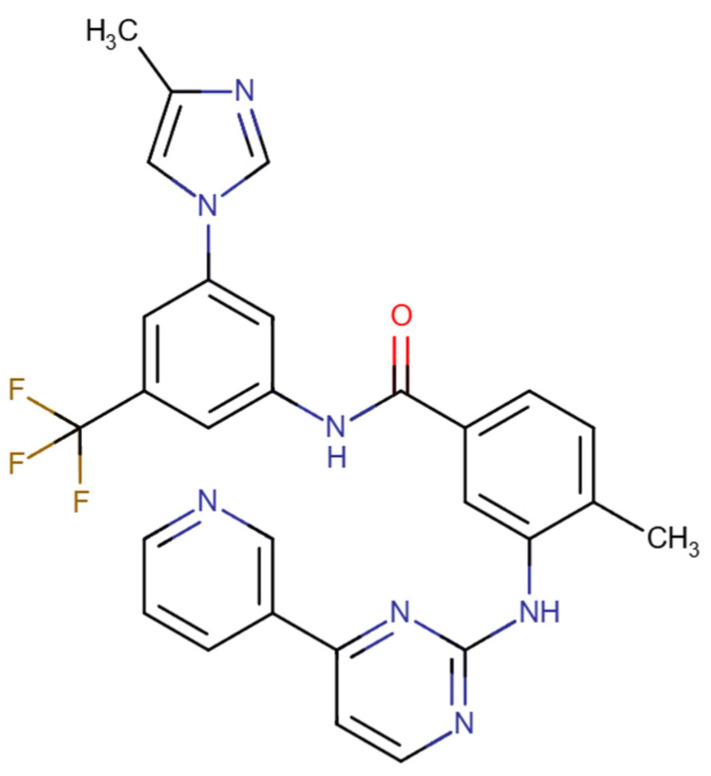
Chemical structures of nilotinib. Available online: https://go.drugbank.com/ (accessed on 21 July 2025).

Nilotinib (Figure 7) is a second-generation Bcr-Abl1 tyrosine kinase inhibitor with higher potency and selectivity for the Abl kinase than imatinib. In CML cell lines that respond to imatinib, nilotinib is approximately 20–50 times more active, whereas in imatinib-resistant cell lines, its activity is 3–7 times greater. It also maintains effectiveness against most imatinib-resistant cell lines carrying Abl kinase mutations. Nilotinib is approved for the treatment of chronic-phase CML as well as for patients with accelerated-phase CML who are intolerant or resistant to imatinib, with a recommended dose of 400 mg twice daily [78,79]. However, despite its high selectivity, nilotinib can cause side effects such as headaches, skin rashes, transient increases in indirect bilirubin, elevated blood glucose levels, and pancreatitis. These adverse effects may require dose reduction, which can usually be performed without significantly reducing the drug’s effectiveness [80]. 

Nilotinib is currently being investigated for repurposing to determine its potential therapeutic benefits in other conditions, particularly in tumours with KIT or PDGFR alterations, including gastrointestinal stromal tumours (GIST) and melanoma, where early studies have shown encouraging results [81,82,83,84]. Studies are also exploring the potential therapeutic role of nilotinib in lung cancer. For the first time, a study evaluated a dual EGFR-TKI approach, combining gefitinib (an EGFR inhibitor) with nilotinib, in patients with EGFR-mutated lung adenocarcinoma and Bcr-Abl1-positive CML. Although the combination was administered safely, extra research is required to better establish the safety and efficacy of nilotinib [85]. In addition, a phase I study evaluating the combination of nilotinib and paclitaxel in patients with advanced solid tumours, including NSCLC, established a maximum tolerated dose—300 mg nilotinib twice daily with 80 mg/m^2^ paclitaxel—and demonstrated promising tolerability [86].

### 3.4. PP2

**Figure 8:**
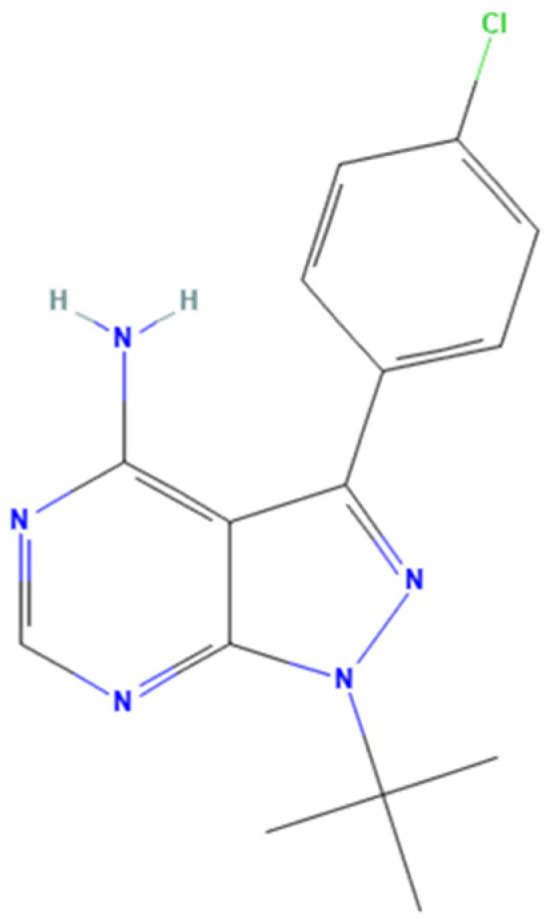
Chemical structures of PP2. Available online: https://pubchem.ncbi.nlm.nih.gov/ (accessed on 9 September 2025).

4-amino-5-(4-chlorophenyl)-7-(dimethyl-ethyl)pyrazolo[3,4-d] pyrimidine (PP2) (Figure 8) is an inhibitor of the Src kinase family, being a selective inhibitor of c-SRC on head and neck squamous cell carcinoma (HNSCC), with an in vitro IC_50_ of 300 nM. Moreover, this drug appears to be highly effective in blocking Lck (IC_50_ = 4 nM) and Fyn (IC_50_ = 5 nM) [87,88]. PP2 has been studied in cancer, showing good anti-tumour activity, anti-oxidative stress, and anti-inflammatory effects [89]. In addition, PP2 appears to be highly useful in preventing cancer metastasis, as it can activate the E-cadherin-mediated cell adhesion system, which plays a key role in metastasis [90].

Despite PP2 still not being approved for any cancer treatment, there are several studies investigating the efficacy of this drug in tumour control, such as in HNSCC, and breast cancer, more specifically, triple-negative breast cancer (TNBC). In those cancers, PP2 effectively blocked cell migration and EMT [87,91,92]. Combination studies were also performed with PP2. In colon cancer, the combination of PP2 with a urokinase-type plasminogen activator receptor (u-PAR) antagonist reduced cancer cell invasiveness [93]. Another study showed that PP2 in combination with all-trans-retinoic acid and arsenic trioxide improved differentiation of acute promyelocytic leukemia (APL) cells [94]. Conversely, in malignant glioma cells, the combination of PP2 with temozolomide did not result in a significant reduction in tumour growth [95].

In lung cancer, efforts have been made to understand the therapeutic efficacy of PP2. Studies in A549 cells have shown that this drug can inhibit cell viability, migration, and invasion by upregulating the expression level of Cx43 protein and suppressing the PI3K/Akt signalling pathway. Furthermore, it induces apoptosis in cancer cells by modulating the PI3K/Akt/Bcl-2/caspase-3 signalling pathway [96,97]. 

### 3.5. Tirbanibulin

**Figure 9:**
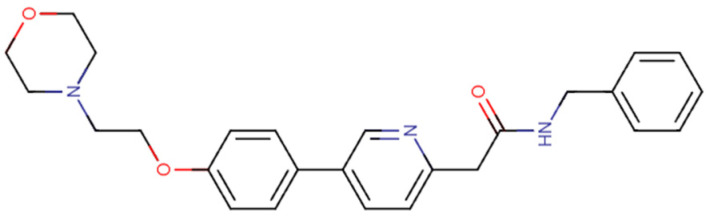
Chemical structures of tirbanibulin. Available online: https://go.drugbank.com/ (accessed on 21 July 2025).

Tirbanibulin (Figure 9), approved for the topical treatment of actinic keratoses—precancerous skin lesions on the face and bald scalp—is a reversible tubulin-binding agent, interfering with microtubule formation and inducing a complete cell cycle arrest at G2/M phase, leading to the activation of intrinsic and extrinsic apoptotic pathways. Studies have already shown the high affinity and specificity of tirbanibulin to tubulin, causing an anti-proliferative effect and stopping cell division. Interestingly, studies proved that Src activity is linked to microtubule (MT) dynamics, as MTs regulate Src intracellular trafficking through FAK, integrins, Rho, and GEF-H1 signaling. Thus, MT-disrupting agents like colchicine or paclitaxel suppress FAK/Src phosphorylation, reducing cancer cell invasion. Tirbanibulin is a distinctive agent because it both binds Src directly and inhibits MT polymerization, potentially performing a synergistic suppression of Src signaling. Nonetheless, the relative contribution of each mechanism to the anticancer effect remains unclear. Additionally, tirbanibulin can increase the expression of the tumour suppressor p53, which contributes to an anti-proliferative effect through multiple pathways [98,99,100]. In vitro studies using human keratinocytes have reported an IC_50_ of 32 nmol/L [101,102].

Although tirbanibulin is currently approved mainly for topical use, early-phase clinical trials have explored its oral administration to evaluate efficacy in several cancers, including acute myeloid leukemia, as well as prostate, breast, pancreatic, colon, ovarian, and lung cancers. In vitro trials showed that tirbanibulin achieved a 50% maximal inhibition of cell growth (GI_50_) of 23 nM against the highly active Src colon cancer cell line HT29 [101,103]. Moreover, in vitro studies have demonstrated that tirbanibulin exhibits potent antiproliferative activity across a variety of cancer cell lines. Among renal cancer cell lines, GI_50_ values ranged from 33 to 45 nM, while non-Hodgkin lymphoma cell lines showed even greater sensitivity, with GI_50_ values between 14 and 19 nM. Melanoma cell lines exhibited GI_50_ values from 21 to 38 nM, and squamous cell carcinoma (A431) demonstrated a GI_50_ of 15 nM. Gastric cancer cell lines had a larger range of sensitivity, with GI_50_ values crossing from 16 to 105 nM. In addition, tirbanibulin showed activity against multi-drug-resistant tumour models, including uterine sarcoma (GI_50_ = 34 nM) and ovarian cancer (GI_50_ = 56 nM) [100].

## 4. Lung Cancer

Lung cancer is the most common cancer worldwide, is typically diagnosed at advanced stages, and is characterized by poor outcomes [104,105]. Usually, lung cancer is diagnosed among smokers, in approximately 80% of cases. However, the number of cancer cases in non-smokers is increasing [106,107]. The high mortality of lung cancer is emphasized because screening programs are limited across the world, and participation in existing programs is low, resulting in late detection and reduced survival outcomes [108]. Low-dose computer tomography (LDCT) is the recommended exam to perform for lung cancer diagnosis. Due to the high heterogeneity of this cancer, a careful tumour characterization after a positive LDCT is crucial in order to choose the best therapeutic approach [109,110].

Choosing the best therapeutic option is challenging in lung cancer because of its heterogeneity, with numerous molecular profiles and histological subtypes, with NSCLC and SCLC being the most prevalent. This molecular diversity makes lung cancer a promising candidate for personalized medicine and the development of targeted therapies, including the repurposing of existing drugs [110,111,112,113].

As described before, Src and Abl are NRTKs involved in cancer, including in lung cancer. In NSCLC, Src was related to tumour progression, being particularly overexpressed in smokers, appearing in 60–80% of adenocarcinomas and bronchioloalveolar cancers [114]. On the other hand, mutations in either *AbL1* or *Abl2* were identified in lung tumours. An increase in *Abl2* copy number was described in NSCLC cells responsive to dasatinib. Moreover, a rare somatic mutation in *Abl1* was reported in adenocarcinoma, with some of these mutations showing sensitivity to imatinib and dasatinib. Notably, even in lung cancer cells lacking genomic alterations in Abl1 and Abl2, elevated Abl tyrosine phosphorylation has been detected [115]. 

Thus, considering the involvement of Src and Abl in lung cancer, the investigation of inhibitors targeting these NRTKs may represent a valuable therapeutic approach. Based on the evidence discussed above, inhibitors such as saracatinib, imatinib, PP2, nilotinib, and tirbanibulin could serve as promising candidates for drug repurposing in lung cancer, given the studies that have already started exploring their efficacy. Furthermore, integrating personalized medicine by analyzing the genetic and molecular characteristics of lung tumours that are more likely to respond to these drugs could significantly contribute to advancing therapeutic strategies and improving patient outcomes.

## 5. Conclusions

Personalized medicine and drug repurposing have become increasingly relevant in oncology, driven by the development of targeted therapies adapted to the individual characteristics of tumours. Moreover, drug repurposing offers a more cost-effective, safer, and faster alternative for drug development. Nonetheless, for the development of new cancer drugs, it is crucial to understand the signalling pathways and molecular characteristics that are being targeted. Among others, Src and Abl tyrosine kinases play a crucial role in cancer progression by regulating cell proliferation, migration, and angiogenesis. Their inhibition is essential in fighting the tumour. Saracatinib, imatinib, PP2, nilotinib, and tirbanibulin are Src and/or Abl inhibitors that are already approved or under investigation in several cancers, having the potential to be repurposed in other cancer types. Given the involvement of Src and Abl in lung cancer, its heterogeneity, and the suitability of these inhibitors for personalized therapy, they hold significant potential for repurposing in this context. Preclinical studies and early-phase clinical trials have already been conducted in lung cancer models, with some results demonstrating encouraging therapeutic potential. Nonetheless, further investigation, particularly in well-stratified patient populations, is essential to validate and fully elucidate the clinical utility of these Src/Abl inhibitors in lung cancer treatment.

## Figures and Tables

**Table 1:** Summary of ongoing and completed clinical trials evaluating repurposed drugs across various cancer types. The table presents the pharmacological class, drug name, target cancer type, and clinical trial phase. Identification by the ClinicalTrials.gov Identifier is represented [19].

Pharmacological Class	Drug	Repurposing Target (Cancer Type)	Clinical Trial Phase
Anti-Platelet	Aspirin	Colon Cancer	Phase III (NCT02301286)
Anti-Diabetic	Metformin	Glioma	Phase II (NCT05929495)
	Pioglitazone	Head and Neck Cancer	Phase II (NCT00099021)
Vasodilator	Minoxidil	Epithelial Ovarian Cancer	Phase II (NCT05272462)
Anti-Viral	Ribavirin	Acute Myelocytic Leukemia	Phase II (NCT00559091)
Angiotensin Receptor Blocker	Losartan	Pancreatic Cancer	Phase II (NCT01821729)
Beta Blockers	Propranolol	Breast Cancer	Phase II (NCT02596867)
Calcium Channel Blockers	Mibefradil	High-Grade Glioma	Phase I (NCT01480050)
Antibiotic	Minocycline (Tetracycline)	Recurrent Glioma	Phase Ib/II (NCT01580969)
	Tigecycline (Tetracycline)	Acute Myeloid Leukemia	Phase I (NCT01332786)
Disease-Modifying Antirheumatic Drug	Auranofin	Non-Small-Cell Lung Cancer or Small-Cell Lung Cancer	Phase I/II (NCT01737502)
		Chronic Lymphocytic Leukemia	Phase I/II (NCT01419691)

## Data Availability

Not applicable.

## Abbreviations

The following abbreviations are used in this manuscript:

AI	Artificial Intelligence
CML	Chronic myeloid leukemia
DDR	DNA damage response
EMT	Epithelial–mesenchymal transition
FDA	US Food and Drug Administration
GI_50_	Maximal inhibition of cell growth
IC_50_	Half maximal inhibitory concentration
LDCT	Low-dose computer tomography
NRTKs	Non-receptor tyrosine kinases
NSCLC	Non-small cell lung cancer
PM	Personalized medicine
PTKs	Protein tyrosine kinases
RTKs	Receptor tyrosine kinases
SCLC	Small cell lung cancer
SFKs	Src family of protein tyrosine kinases
